# Left-Handedness

**Published:** 1887-01

**Authors:** 


### Laft-Handedness.

A popular English novelist has written an essay, in which he maintains that man is working great injury to himself by the persistent habit which he has of dextral preference, and urges a return to the ambidexterity of the primitive races. In Franklin's well-known "Petition of the Left Hand,"the same idea is also expressed. But despite the arguments and pleas of these and other writers, ninety-one per cent. of civilized beings continue to use their right arms and hands in preference to their left, and in all probability this proportion will increase rather than diminish in succeeding ages.

Dr. Louis Jobert has published an interesting work on the cause and frequency of left-handedness, in which he has collected a number of curious facts concerning this abnormal preference. No purely left-handed race of men has ever been discovered, yet among certain peoples the proportion of those who use the left-hand in preference to the right is very large. Among the inhabitants of Pendjab, for example, nearly seventy per cent. use the left had by preference. The Hottentots and the Bushmen of South Africa are said also to be, for the greater part, left-handed.

Dextral preference would seem to be a mark of racial, though, of course, not always of individual, superiority. "Man," says Moilin, "is the most asymmetrical of animals as he is the most perfect." The new-born child uses one hand as well as the other, but as his faculties become developed he begins to show a preference for one or the other hand, and usually for the right. One of the curious results of the study of crime, which is pursued so industriously by the Italian physicians, is the discovery that left-handedness is very common among criminals. Dr. Marro found that from fourteen to over twenty-two per cent. of convicted criminals were left-handed, while the highest ratio among people of all classes is only about nine to one hundred. Among the insane also dextral preference is found to be much less marked.

It would thus appear that, while ambi-dexterity is theoretically an advantage, it is not to be expected that the human race will ever acquire it, since the tendency in evolution is to differentiation of the two hands, just as it was formerly to differentiation of the upper from the lower extremities. Right-handedness is the price which we have to pay for a higher civilization and greater mental acquirements; while left-handedness would seem to be a step backward and a tendency to a reversal toward primitive man.

